# Seroprevalence and Risk Factors Possibly Associated with Emerging Zoonotic Vaccinia Virus in a Farming Community, Colombia

**DOI:** 10.3201/eid2512.181114

**Published:** 2019-12

**Authors:** Ashley Styczynski, Jillybeth Burgado, Diana Walteros, José Usme-Ciro, Katherine Laiton, Alejandra Pinilla Farias, Yoshinori Nakazawa, Christina Chapman, Whitni Davidson, Matthew Mauldin, Clint Morgan, Juan Martínez-Cerón, Edilson Patiña, Leidy Laura López Sepúlveda, Claudia Patricia Torres, Anyely Eliana Cruz Suarez, Gina Paez Olaya, Carlos Elkin Riveros, Diana Yaneth Cepeda, Leydi Acosta Lopez, Daniela Gomez Espinosa, Faiber Antonio Gutierrez Lozada, Yu Li, P.S. Satheshkumar, Mary Reynolds, Martha Gracia-Romero, Brett Petersen

**Affiliations:** Centers for Disease Control and Prevention, Atlanta, GA, USA (A. Styczynski, J. Burgado, Y. Nakazawa, C. Chapman, W. Davidson, M. Mauldin, C. Morgan, Y. Li, P.S. Satheshkumar, M. Reynolds, B. Petersen);; Instituto Nacional de Salud, Bogotá, Colombia (D. Walteros, J. Usme-Ciro, K. Laiton, A.P. Farias, M. Gracia-Romero);; Universidad Cooperativa de Colombia, Santa Marta, Colombia (J. Usme-Ciro);; Universidad de Antioquia, Medellín, Colombia (J. Martínez-Cerón, E. Patiña, L.L. López Sepúlveda);; Secretaria de Salud de Cundinamarca, Bogotá (C.P. Torres, A.E. Cruz Suarez, G.P. Olaya, C.E. Riveros);; Hospital Nuestra Señora del Pilar de Medina, Medina, Colombia (D.Y. Cepeda);; Vigilancia en Salud Publica, Bogotá (L.A. Lopez);; Universidad Javeriana, Bogotá (D.G. Espinosa);; Secretaria de Salud de Caquetá, Florencia, Columbia (F.A. Gutierrez Lozada)

**Keywords:** vaccinia virus, orthopoxvirus, zoonotic, dairy farms, Colombia, viruses, zoonoses

## Abstract

In 2014, vaccinia virus (VACV) infections were identified among farmworkers in Caquetá Department, Colombia; additional cases were identified in Cundinamarca Department in 2015. VACV, an orthopoxvirus (OPXV) used in the smallpox vaccine, has caused sporadic bovine and human outbreaks in countries such as Brazil and India. In response to the emergence of this disease in Colombia, we surveyed and collected blood from 134 farmworkers and household members from 56 farms in Cundinamarca Department. We tested serum samples for OPXV antibodies and correlated risk factors with seropositivity by using multivariate analyses. Fifty-two percent of farmworkers had OPXV antibodies; this percentage decreased to 31% when we excluded persons who would have been eligible for smallpox vaccination. The major risk factors for seropositivity were municipality, age, smallpox vaccination scar, duration of time working on a farm, and animals having vaccinia-like lesions. This investigation provides evidence for possible emergence of VACV as a zoonosis in South America.

## Abstract 2

Dairy farmers had high rates of orthopoxvirus seropositivity and substantial illness associated with vaccinia-like lesions.

Vaccinia virus (VACV) is a member of the genus *Orthopoxvirus* within the family *Poxviridae*. Other notable viruses in this lineage include cowpox, monkeypox, and variola (causative agent of smallpox). Because of immunologic cross-reactivity of orthopoxviruses (OPXVs), cutaneous inoculation with VACV through a worldwide vaccination campaign led to the eradication of smallpox in 1980. However, unlike variola virus, VACV can infect nonhuman hosts (*1*). The origin of VACV remains unknown, but the virus is thought to have originated in continental Europe before being isolated and used as the vaccine against smallpox (*2*). Transmission of VACV from humans to cattle was reported during the smallpox eradication campaign, which has engendered debate over whether VACV escaped into animals as a result of vaccination efforts (*3*–*9*). Regardless of the event that led to zoonotic circulation, recent studies have demonstrated ongoing infections with related VACV viruses in Brazil, suggesting endemic spread through a common reservoir (*10*,*11*).

Several sporadic outbreaks of VACV have been reported in humans and cattle in Brazil and India, where mechanisms of transmission have been attributed to cross-inoculation between teats of cows and hands of milkers (*12*–*19*). Although no reservoir has been identified, data suggest that rodents might be implicated in the transmission and maintenance of the virus (*4*,*20*–*23*). Furthermore, laboratory studies have demonstrated the feasibility of rodents as reservoirs (*20*,*24*,*25*).

VACV outbreaks have proven hazardous in terms of human health and economic impact (*12*,*18*), but without an identifiable reservoir, control efforts are limited to hygiene and isolation strategies. In addition, prior smallpox vaccination is not necessarily protective against VACV during outbreaks, likely because of waning immunity (*17*). Another potential concern is the transmission of VACV through the milk of affected cows, which has been experimentally demonstrated by the persistence of viable virus despite heat or refrigeration (*26*–*30*).

In the course of increased surveillance and education activities, Colombia has confirmed VACV infections in >3 departments; several additional cases of similar pox-like lesions have been reported throughout the country, particularly affecting farmworkers responsible for milking cows (Andres Paez, Instituto Nacional de Salud, pers. comm., email, 2015 Oct 7). Phylogenetic analyses of isolates obtained from case-patients in Colombia demonstrate some differences from strains circulating in Brazil, although limited genetic sequencing precludes definitive determination of the source (*31*,*32*). This genetic divergence suggests that VACV might be widespread in Colombia; however, its distribution and associated risk factors for transmission have not been systematically evaluated. To help clarify the burden of VACV and risk factors associated with disease exposure, we conducted a serosurvey and risk factor assessment in the municipality of Medina in Cundinamarca Department, Colombia, where several human cases of PCR-confirmed VACV infections had been reported in the preceding year.

## Methods

### Respondent Selection

During August–September 2016, we performed a serologic investigation of farmworkers and household members in Cundinamarca Department. We selected farms based on respondent availability from a list of farms provided by the local secretary of health. After obtaining informed consent from adults and permission from parents of children <18 years of age, we administered a questionnaire regarding demographic characteristics, exposures, travel history, and farming practices. We also collected serum samples from interviewees to correlate risk factors with serologic evidence of VACV exposure. We received a total of 134 responses and corresponding specimens from persons on 56 separate farms.

### Orthopoxvirus Antibody Detection

We used IgG ELISA to evaluate the presence of orthopoxvirus-specific antibodies (i.e., anti-OPXV) as previously described (*33*). We coated Immulon II High Binding microtiter plates (ThermoFisher Scientific, https://www.thermofisher.com) with purified VACV DryVax strain at 0.1µg/mL in carbonate buffer, incubated overnight at 4°C, inactivated with 10% formalin, and washed 3 times with PBST (PBS with 0.05% Tween-20) by using a BioTek 405TS plate washer (Biotek, https://www.biotek.com). We then blocked plates at room temperature for 30–60 min with assay diluent containing 5% dried skim milk, 2% normal goat serum, and 2% bovine serum albumin in PBST. After blocking, we washed plates 3 times with PBST, added serum samples at 1:100 dilution in duplicate, and incubated for 1 h at 37°C. We washed plates again and added goat anti-human IgG horseradish peroxidase conjugate (KPL antibodies) (SeraCare, https://www.seracare.com) at 1:2,000 concentration, incubated for 1 h at 37°C, and washed. We then added SureBlue TMB 1-component microwell peroxidase substrate (KPL antibodies) (SeraCare) and developed for 4 min at room temperature before stopping the reaction with addition of equal volume of TMB Stop Solution (SeraCare). We read optical density (OD) on an Enspire plate reader (Perkin Elmer, https://www.perkinelmer.com) at 450 nm.

For the IgM ELISA, we coated microtiter plates (Immulon II) with goat anti-human IgM KPL antibodies at 1:800 dilution in PBS (pH 7.4) and incubated overnight at 4°C. We then washed plates 5 times with PBST by using a plate washer and blocked for 30 min to 1 h at room temperature with assay diluent buffer containing 0.5% gelatin, 2% BSA, 5% skim milk, and 2% normal goat serum in PBST. We added test serum samples at 1:50 dilution in duplicates in assay diluent and incubated for 1 h at 37°C. We washed plates, added antigen (purified VACV) at a concentration of 0.5 µg/mL, and incubated for 1 h at 37°C. We washed plates again and incubated with 1:250 dilution of anti-variola virus hyperimmune mouse polyclonal ascetic fluid for 1 h at 37°C, followed by washing and incubation with 1:6,000 dilution of goat anti-mouse IgG horseradish peroxidase conjugate (KPL antibodies) for 1 h at 37°C. We then washed the plates again and developed with SureBlue TMB 1-component microwell peroxidase substrate for 8 min at room temperature, after which we added equal volume of TMB Stop Solution to each well. We read on an Enspire plate reader at 450 nm.

We averaged OD values for known negative controls and determined a cutoff value by using the equation cutoff value: average negatives + 3 × SD of negatives. We subtracted the cutoff value from the OD values of test samples. If the resulting value was >0.05, we considered the serum sample positive for the presence of OPXV antibodies.

### Data Analyses

To identify risk factors associated with OPXV exposure, we performed a nested case–control analysis on the basis of serologic test results. We classified as case-patients those persons with a positive test for OPXV IgM or IgG, which is not specific for VACV but is a reasonable approximation of exposure (either through natural infection or vaccination), given a lack of other known circulating OPXVs in this region. Conversely, we identified as controls those persons without serologic evidence of OPXV exposure. To determine odds ratios (ORs) and 95% CIs, we performed a complex sample analysis to account for clustering of responses and serologic outcomes by farm. Variables found to have a p value <0.1 in bivariate analysis were included in a multivariable model analysis.

We also evaluated the correlation of farm-level characteristics with seropositivity of any persons associated with the farm. We performed bivariate analysis on individual risk factors to determine ORs and 95% CIs. Given that the last smallpox vaccination campaign occurred in Colombia in 1972, we separated persons on the basis of age of eligibility to have received the smallpox vaccine (*34*).

We subsequently built 2 multivariate logistic regression models by using individual-level risk factor data and farm survey data, respectively. We used seropositivity as the outcome variable. Using simple logistic regression, we included all variables found to be statistically significant at an α level of 0.1 in a stepwise model selection procedure. For individual-level risk factor data, we incorporated the variable that was most significant after being solely added to the model (if any were significant at an α level of 0.1) into the model. If any of the tested variables were no longer significant after this addition at an α level of 0.1, we dropped it from the model. This process continued until no variable was found to be significant, after each was solely added to the model. We checked variables for collinearity by using Pearson correlation coefficients; values <0.4 were considered to not be collinear.

We conducted a similar process with the farm survey data. In that case, we also forced into the model the variable indicating whether any animals with a history of vaccinia-like lesions were on the farm. We did this to evaluate the influence of suspected animal vaccinia virus infections on human seropositivity. Afterward, we conducted the same step-wise procedure.

### Ethics Statement

Review by the Colombian Instituto Nacional de Salud and a human subjects advisor at the US Centers for Disease Control and Prevention determined that the activities did not meet the definition of research under 45 CFR 46.102(d). All adult participants provided informed written consent before interview participation and collection of specimens. Participants <18 years of age provided age-appropriate assent, and parents or guardians provided consent on their behalf.

## Results

### Demographics and Descriptive Analysis

Commensurate with the agricultural setting that characterizes Cundinamarca Department, participants in the investigation tended to live in rural environments and had frequent contact with animals (Table 1). Approximately equal numbers of men and women were enrolled; median age was 46 years. Based on the given age threshold (44 years of age), slightly more than half of the participants (57%) would have been eligible to have received a smallpox vaccine before the end of the vaccination campaign. However, only 34% of participants recalled a history of smallpox vaccination. 

**Table 1:** Demographic characteristics of 134 farmworkers and household members from 56 farms in Cundinamarca Department, Colombia, August–September 2016*

Characteristic	Value
Sex	
M	69 (51.5)
F	65 (48.5)
Median age, y (range)	45.5 (12–82)
Municipality of residence	
Medina	114 (85.1)
Ubala	19 (14.2)
Paratebueno	1 (0.7)
Education	
None	25 (18.7)
Primary	67 (50)
Secondary	22 (16.4)
Post-secondary	9 (6.7)
Other	11 (8.2)
Live in rural setting	128 (95.5)
Work outdoors	125 (93.3)
Work with animals	128 (95.5)
Self-report history of smallpox vaccination	46 (34.4)
Eligibility for smallpox vaccine (i.e., respondents age >44 y)	76 (56.7)
Seropositivity	
IgM	2 (1.5)
IgG	70 (52.2)
IgM or IgG	70 (52.2)
IgM or IgG among respondents age <44 y	18 (31)
Contact with cows	129 (96.3)
Milk cows	115 (85.8)
Work on multiple farms concurrently	50 (37.3)
Prior knowledge of poxviruses	28 (20.9)

*Values are no. (%) persons except as indicated.

Nearly all participants (96%) reported contact with cows, and most of these persons participated in the milking process (86%). Thirty-seven percent of participants reported working concurrently on multiple farms, and ≈21% of participants had previously heard of VACV or other poxviruses.

Laboratory analysis demonstrated that 70 (52%) of the 134 participants were OPXV IgG positive, including 2 (1.5%) persons who were also OPXV IgM positive, suggesting a recent exposure (<6 months before). None of the participants was only positive for IgM. Excluding those born in 1972 or earlier, seropositivity for OPXV IgM or IgG was found among 18 (31%) of 58 people included in this category (Appendix Figure 1).

Eighteen seropositive persons also reported a history of a vaccinia-like lesion, primarily occurring on the hand (94.4%), but 3 persons reported eye involvement (Table 2). Of these 18 persons, 12 (67%) were <44 years of age, making them ineligible to have received a smallpox vaccine. The risk for symptomatic vaccinia-like lesions was not statistically different between those who self-reported smallpox vaccination and those who did not recall a history of vaccination (OR 0.35, 95% CI 0.1–1.3). However, when we compared age of smallpox vaccine eligibility, being <44 years of age was strongly correlated with having a symptomatic vaccinia-like infection (OR 15.3, 95% CI 4.2–56.1).

**Table 2:** Characteristics of 18 OPXV-seropositive persons with history of vaccinia-like lesions among farmworkers and household members from 56 farms in Cundinamarca Department, Colombia, August–September 2016*

Characteristic	Value
Age <44 y	12 (66.6)
Location of lesion(s)†	
Hand	17 (94.4)
Eyes	3 (16.7)
Arm	1 (5.6)
Face or neck	1 (5.6)
Leg	0
Median no. lesions (range)	1.5 (1–6)
Prior injury at site of lesion	5 (27.8)
Residual scar	13 (72.2)
Time off work because of lesion(s), d	10 (55.6)
Median time off work, d (range)	10 (3–15)
Evaluated by physician	11 (61.1)
Hospitalized	2 (11.1)
Lesion symptoms	
Localized pain	18 (100.0)
Pruritus	17 (94.4)
Swelling	15 (83.3)
Warmth	14 (77.8)
Discharge	12 (66.7)
Lymphangitis	11 (61.1)
Constitutional symptoms	
Generalized pain	10 (55.6)
Headache	10 (55.6)
Fever after lesion	10 (55.6)
Fever before lesion	4 (22.2)
Chills or rigors	4 (22.2)
Lymphadenopathy	3 (16.7)
Arthralgias	1 (5.6)
Myalgias	1 (5.6)

*Values are no. (%) persons except as indicated. OPXV, orthopoxvirus. †Number of lesion locations is >18 because some persons had >1 lesion.

Symptomatic persons experienced a median of 1.5 lesions, and lesions resulted in scarring in 13 of the 18 patients. Approximately one half of these persons took time off work because of their lesions, for a median of 10 days (range 3–15 days). Eleven people sought care from a physician, and 2 persons were hospitalized.

The lesions were most frequently characterized by localized pain and swelling, pruritus, and increased warmth (Table 2). Two thirds of patients also reported discharge from the lesion and lymphangitis. Many of the patients cited the co-occurrence of other symptoms including fever, malaise, and headache.

In the analysis of farm-level characteristics, 22 (39%) of the 56 farms reported animals with vaccinia-like lesions. Cows were the only domesticated animals noted to have vaccinia-like lesions, with the exception of 1 farm that also recalled pigs having similar lesions. The lesions were located on the udders or teats in all cases; 2 farms also reported oral lesions, and 1 farm reported genital lesions. Twenty (91%) of the 22 farms continued milking their cows in spite of the lesions. Outcomes of the lesions resulted in decreased milk production at 5 farms and caused scarring of the affected cows at 3 farms.

### Bivariate Analysis

In the bivariate analysis of individual-level risk factors that we assessed, 13 variables were significantly associated with anti-OPXV seropositivity, including age as a continuous variable or as a dichotomous outcome based on eligibility for smallpox vaccination (Appendix Table 1). Age (dichotomous) and consumption of pork were the variables most strongly associated with seropositivity (OR 4.81 for age, OR 4.86 for pork consumption). Among the other significant factors were municipality of residence, self-reported history of smallpox vaccination, presence of smallpox vaccination scar, cows living on the property of residence, time spent working on the current farm, previous work on other farms, and consumption of unpasteurized milk or cheese.

In the evaluation of farm-level risk factors, 7 variables were associated with seropositivity among farmworkers at the 0.1 level (Appendix Table 2). These variables included animals with vaccinia-like lesions, type of cattle feed, habitats surrounding the farm, and humans on the farm with vaccinia-like lesions.

### Multivariate Analysis

For the multivariate analysis of individual-level risk factors, 5 variables were included in the final model: age (dichotomous), smallpox vaccination scar, in-country travel in the previous 12 months, duration of time spent working on current farm, and municipality of residence. All variables were significant at the p<0.05 level, and none were found to be collinear. Age >44 years, presence of a vaccination scar, and longer duration of time working on the current farm were predictive of anti-OPXV seropositivity, whereas in-country travel and residence outside of Medina were protective (Table 3).

**Table 3:** Multivariate analysis of OPXV IgM or IgG seropositivity among farmworkers and household members from 56 farms in Cundinamarca Department, Colombia, August–September 2016*

Variable	OR (95% CI)	p value
Individual-level risk factors		
Age (dichotomous)	3.38 (1.31–8.74)	0.01
Smallpox scar	5.18 (1.71–15.66)	<0.01
In-country travel	0.11 (0.03–0.42)	<0.01
Duration of time working at current farm	2.34 (1.03–5.30)	0.04
Residence other than Medina	0.26 (0.07–1.04)	0.01

Farm-level risk factors		
Animals with vaccinia-like lesions	5.71 (0.90–36.19)	0.06
Commercial feed	0.16 (0.03–0.83)	0.03
Cattle fed after milking	0.19 (0.03–1.15)	0.07

*OPXV, orthopoxvirus; OR, odds ratio.

Farm-level risk factors in the final model included animals with a history of vaccinia-like lesions, use of commercial feed, and feeding cattle after milking. Variables were significant at the p<0.1 level. Animals having vaccinia-like lesions was predictive of anti-OPXV seropositivity of farmworkers, but the other 2 variables were noted to be protective (Table 3).

## Discussion

VACV is probably an emerging zoonosis in Colombia and poses a substantial health risk for the populations affected; namely, farmworkers involved in the dairy industry. In this investigation, OPXV seropositivity along with vaccinia-like symptoms among farmworkers resulted in increased use of healthcare services, loss of productive work days, and dermatologic scarring at the sites of infection. VACV-like infections among cattle resulted in decreased milk production and permanent scarring of teats.

Descriptions of VACV-like infections in this population revealed mostly localized, painful, cutaneous lesions affecting the hands, similar to other descriptions of bovine-related VACV infections (*13*,*17*,*35*). More than half of the patients also reported accompanying systemic symptoms such as fevers and malaise, and most of those affected required medical attention and time off work, indicating substantial economic ramifications. In addition, two thirds of the persons who were seropositive and reported a history of symptomatic lesions were ineligible to have received a smallpox vaccine, supporting the idea that unvaccinated persons are at greater risk for symptomatic disease (*12*).

Regarding individual-level risk factors, the association of age and smallpox vaccination scar with OPXV seropositivity is expected because these are proxy (albeit imperfect) measures of smallpox vaccination status. Rural areas of the country might have ceased smallpox vaccination before 1972, and smallpox vaccination scars can be confused with bacillus Calmette–Guérin vaccination scars. As such, the actual effect of age on VACV exposure cannot be determined. Increased age might reflect a greater opportunity for exposure, which might explain the correlation with longer duration of working on the current farm, although this correlation might not be relevant if VACV only recently emerged in Colombia. More important, nearly one third of participants who were seropositive would have been ineligible for smallpox vaccination, signifying ongoing risk for population transmission (*36*).

Medina was the center of the VACV outbreak; therefore, living in Medina would be expected to be associated with seropositivity. However, because our investigation was geographically centered on Medina, very few participants resided outside this municipality. A more extensive investigation of other dairy-producing areas in the country might reveal differing results. The finding that in-country travel was protective might suggest that VACV is not extensively circulating in other areas of Colombia.

The reasons for consumption of pork strongly being correlated with seropositivity in the univariate analysis are not clear, given that pigs are not known to be natural hosts of VACV. In addition, few farms in this investigation raised pigs, although nearly all participants reported consuming pork. The fact that 1 farm did report vaccinia-like lesions on pigs might warrant further investigation using PCR testing. Regardless, this variable was excluded through stepwise selection in the multivariate analysis, possibly indicating a measure of confounding.

Among farm-level characteristics, the correlation of human seropositivity with animals having vaccinia-like lesions demonstrates that farmers correctly identified lesions on cattle as being consistent with VACV, although this finding does not answer the question of whether cattle acquired the infection from milkers or vice versa. The observed protective effect of commercial feed might be attributable to commercial feed being less likely to be contaminated by rodent urine and feces, which have been shown to harbor VACV (*24*,*25*). Reduced VACV exposure by cattle would thus translate into reduced human exposure. 

Variables that do not correlate with seropositivity might be as informative as variables that predict seropositivity. In particular, having rodents near the residence, having other household members with VACV-like lesions, consuming unpasteurized dairy products, and having cows that live on the property were not associated with seropositivity in multivariate analysis. These findings underscore that humans are more frequently infected through interaction with cows than with rodents.

VACV has been documented to spread within households, including through household fomites (*31*,*37*,*38*), so it is somewhat surprising that having other household members with VACV-like lesions was not identified as a risk factor in this investigation. This finding might indicate that household transmission is not a primary mechanism of VACV spread and that the main means of transmission might be directly from cows to humans. Alternatively, because a high rate of respondents had contact with cows, the significance of transmission only through household contact could not be elucidated. Furthermore, an average of only 2 persons from most households participated in interviews and blood sample collection, so a more dedicated investigation might be needed to evaluate the significance of household spread.

VACV has been detected in unpasteurized dairy products (*24*), but the effect of such contamination on VACV transmission is unknown. In our investigation, consuming unpasteurized dairy products did not correlate with seropositivity, which might indicate that such consumption is not an important mechanism for VACV exposure. Nonetheless, additional population-level studies and testing of dairy products should be performed before negating the consumption of unpasteurized dairy products as a potential risk factor, especially given the high rate of farms that continued milking their cows despite the presence of active lesions. Further assessments regarding dairy products as a potential mechanism of disease spread will be necessary for guiding public health recommendations.

Despite an extensive questionnaire, few farming practices were found to correlate with human seropositivity. This finding could indicate that farming practices do not affect VACV transmission, but, more likely, it reflects homogeneity of farming practices that did not enable distinguishing between specific practices. Of note, all of the surveyed farms had small numbers of cattle and performed manual milking, making the risk for contact transmission either between cattle or between humans and cattle particularly germane. Additional investigation regarding animal seropositivity will be important for gaining insight into the effects of farming practices.

The findings of this investigation are similar to results from studies carried out in Brazil that found a positive correlation between age and seropositivity, although the effect of prior smallpox immunization could not be ruled out. In addition, report of animals with a history of vaccinia-like lesions was predictive of human seropositivity (*39*).

Clinical descriptions of painful, cutaneous lesions with associated systemic symptoms of headache, fever, and lymphadenopathy align closely with descriptions from Brazil during bovine-associated human outbreaks. Also similar to previous reports, vaccinia-like lesions were reported among persons who would have been age-eligible and self-reported prior smallpox vaccination, implying that prior vaccination might be only partially protective (*12*,*17*,*35*,*40*).

The results of this investigation offer additional insight on the emergence of bovine-associated VACV-like infections in Colombia, which has only recently been described. OPXV seropositivity was linked to VACV-like symptoms in 13% of persons, particularly among those who had not been vaccinated against smallpox, demonstrating a substantial burden of disease in this population. However, these results do not provide a full understanding of the geographic extent of VACV circulation in Colombia, and more widespread assessments that include PCR data will be important for estimating population-level effects.

This outbreak investigation reveals that VACV is likely to become an increasingly important zoonosis in this part of the world, either through independent emergence events or expanding reservoir habitats against a backdrop of waning immunity. Using this type of data to clarify risk factors associated with seropositivity and disease transmission, alongside models that predict areas of disease spread, will be important for directing public health efforts to raise awareness and implement preventive measures to minimize adverse social and economic effects (*36*,*41*).

Appendix Additional information regarding seroprevalence and risk factors possibly associated with emerging zoonotic vaccinia virus in a farming community, Colombia.
