# Supplementary material for: Seroprevalence and Risk Factors Possibly Associated with Emerging Zoonotic Vaccinia Virus in a Farming Community, Colombia
Source: Emerg Infect Dis. 2019 Dec;25(12):2169–76. doi: 10.3201/eid2512.181114 (PMC6874243; doi:10.3201/eid2512.181114)
Supplement: Appendix — Additional information regarding seroprevalence and risk factors possibly associated with emerging zoonotic vaccinia virus in a farming community, Colombia. [file 18-1114-Techapp-s1.pdf]

Article DOI: https://doi.org/10.3201/eid2512.181114

Seroprevalence and Risk Factors Possibly Associated with Emerging Zoonotic Vaccinia Virus in a Farming Community, Colombia

Appendix

Appendix Table 1. Bivariate individual-level risk factor analysis of anti-OPXV (IgM or IgG) seropositivity

Variable	Unadjusted OR (95% CI)	Adjusted OR (95% CI)*	p-value
Age, y			
<44	Reference		
≥44	4.81 (2.34–10.26)	4.81 (2.32–10.01)	**<0.01**
Age (continuous)	1.04 (1.02–1.07)	1.04 (1.02–1.07)	**<0.01**
Men	1.12 (0.57–2.22)	1.12 (0.62–2.04)	0.71
Municipality of residence			
Medina	3.22 (1.16–10.43)	3.22 (1.40–7.39)	0.01
Other	Reference		
Housing material (any)			
Brick	0.62 (0.31–1.23)	0.62 (0.31–1.25)	0.19
Adobe	Undefined		
Wood	1.09 (0.55–2.17)	1.09 (0.51–2.33)	0.83
Cement block	1.28 (0.56–3.03)	1.28 (0.56–2.97)	0.56
Concrete	0.71 (0.33–1.54)	0.71 (0.31–1.65)	0.43
Other	1.23 (0.26–6.47)	1.23 (0.27–5.70)	0.79
Live in urban setting	0.44 (0.06–2.34)	0.44 (0.11–1.79)	0.26
Work outside	0.87 (0.21–3.42)	0.87 (0.23–3.23)	0.83
Work with animals	1.10 (0.20–6.13)	1.10 (0.23–5.33)	0.91
Level of education	1.04 (0.89–1.24)	1.04 (0.90–1.21)	0.58
Self-report history of smallpox vaccination	4.20 (1.91–9.65)	4.20 (1.91–9.22)	**<0.01**
Contact with person recently vaccinated against smallpox	1.25 (0.35–4.55)	1.25 (0.39–3.95)	0.71
Presence of smallpox vaccine scar	4.00 (1.71–10.07)	4.00 (1.63–9.86)	**<0.01**
Contact with pets (weekly+)	1.51 (0.82–2.84)	1.51 (0.77–2.97)	0.24
Free-roaming pets	3.59 (0.45–73.67)	3.59 (0.36–36.05)	0.28
Contact with cows (weekly+)	3.45 (0.43–70.75)	3.45 (0.35–33.62)	0.29
Cows living on property of residence	0.42 (0.17–0.98)	0.42 (0.18–0.99)	**0.05**
Participate in milking cows (weekly+)	0.61 (0.18–1.87)	0.61 (0.18–2.00)	0.41
Work on a farm	1.10 (0.33–3.71)	1.10 (0.33–3.72)	0.87
Duration of time working at current farm	1.85 (1.06–3.44)	1.85 (0.99–3.47)	**0.06**
Work on other farms concurrently	1.24 (0.60–2.57)	1.24 (0.61–2.52)	0.56
Previously worked on other farms	1.98 (0.96–4.14)	1.98 (1.03–3.82)	**0.05**
Scratched by an animal	0.52 (0.25–1.06)	0.52 (0.23–1.15)	0.11
Bitten by an animal	1.12 (0.57–2.22)	1.12 (0.60–2.11)	0.72
Sightings of mice or rats in/near the house	0.84 (0.98-undef)	0.84 (0.36–1.97)	0.69
In-country travel within 12 mo	0.33 (0.13–0.79)	0.33 (0.11–1.03)	**0.06**
Ever traveled internationally	0.91 (0.21–4.00)	0.91 (0.28–2.97)	0.88
Gardens	1.74 (0.84–3.67)	1.74 (0.85–3.53)	0.13
Fertilizes with animal manure	2.46 (0.47–14.16)	2.46 (0.51–11.99)	0.28
Handles animal products (e.g., hides, wool, etc.)	0.44 (0.16–1.16)	0.44 (0.16–1.25)	0.13
Hunting within 12 mo	0.60 (0.24–1.45)	0.60 (0.28–1.27)	0.19
Processing of meat within 12 mo	0.99 (0.50–1.95)	0.99 (0.53–1.85)	0.97
Consumption of unpasteurized (raw) milk or cheese	0.55 (0.27–1.10)	0.55 (0.27–1.10)	**0.10**
Previous knowledge of poxviruses	1.00 (0.97–1.03)	1.00 (0.97–1.03)	0.99
Self-report history of vaccinia-like lesion	1.00 (0.97–1.03)	1.00 (0.97–1.03)	0.98
History of contact with vaccinia-like lesion	1.01 (0.98–1.04)	1.01 (0.98–1.03)	0.59
History of accidental needle stick	1.05 (0.98-undef)	1.05 (0.99–1.12)	0.13
History of transfusion with blood product	0.57 (0.18–1.69)	0.57 (0.17–1.91)	0.37
Household member or co-worker with history of vaccinia-like lesion	2.36 (1.03–5.71)	2.36 (1.18–4.74)	**0.02**

Variable	Unadjusted OR (95% CI)	Adjusted OR (95% CI)*	p-value
Contact with farm animals (weekly+)			
Cows	4.60 (0.66–91.31)	4.60 (0.50–42.10)	0.18
Chickens	0.68 (0.27–1.63)	0.68 (0.29–1.61)	0.38
Horses	1.44 (0.53–4.01)	1.44 (0.57–3.64)	0.45
Pigs	1.60 (0.81–3.19)	1.60 (0.75–3.42)	0.23
Sheep	1.39 (0.22–10.80)	1.39 (0.17–11.03)	0.76
Other	2.82 (0.35–57.84)	2.82 (0.28–28.82)	0.39
Contact with wild animals within 12 mo			
Rats	1.45 (0.73–2.89)	1.45 (0.64–3.29)	0.38
Mice	1.33 (0.67–2.64)	1.33 (0.69–2.57)	0.40
Bats	1.31 (0.65–2.67)	1.31 (0.61–2.82)	0.49
Armadillos	1.03 (0.51–2.05)	1.03 (0.55–1.90)	0.93
Monkeys	1.92 (0.62–1.37)	1.92 (0.93–3.95)	**0.08**
Hares	0.47 (0.14–1.44)	0.47 (0.14–1.56)	0.22
Opossums	1.47 (0.61–3.66)	1.47 (0.64–3.41)	0.37
Feral cats	0.91 (0.27–3.05)	0.91 (0.28–2.95)	0.87
Feral dogs	1.36 (0.49–3.97)	1.36 (0.45–4.06)	0.59
Foxes	1.31 (0.56–3.18)	1.31 (0.62–2.79)	0.48
Spent time in various habitats within 12 mo			
Grassland	1.14 (0.54–2.39)	1.14 (0.51–2.52)	0.75
Crop fields	1.81 (0.88–3.77)	1.81 (0.99–3.29)	**0.06**
River	0.82 (0.39–1.71)	0.82 (0.41–1.65)	0.58
Forest	1.47 (0.69–3.16)	1.47 (0.62–3.50)	0.39
Mountains	0.70 (0.31–1.53)	0.70 (0.32–1.53)	0.37
Type of meat consumed			
Beef	0.60 (0.15–2.09)	0.60 (0.15–2.39)	0.47
Pork	4.86 (1.16–33.06)	4.86 (0.96–24.68)	**0.06**
Chicken	1.67 (0.27–13.01)	1.67 (0.29–9.80)	0.57
Fish	3.39 (0.42–69.59)	3.39 (0.33–34.51)	0.31
Lamb	0.91 (0.24–3.41)	0.91 (0.25–3.29)	0.88
Wild game	0.95 (0.48–1.87)	0.95 (0.44–2.04)	0.89
Goat	0.45 (0.02–4.80)	0.45 (0.04–5.09)	0.52

*Adjusted for within-farm correlation using complex sample analysis.

Appendix Table 2. Bivariate farm-level risk factor analysis of anti-OPXV seropositivity

Variable	OR (95% CI)	p-value
Municipality of farm location	Undefined	
Farm size, hectares	1.01 (1.00–1.03)	0.46
Number of personnel	1.05 (0.94–1.42)	0.61
Crops cultivated on the farm		
Corn	2.40 (0.54–16.92)	0.30
Bananas	1.61 (0.48–5.69)	0.44
Sugar	1.88 (0.41–13.36)	0.46
Grass or hay	Undefined	
Other crops	1.96 (0.58–6.95)	0.28
Uncultivated land surrounding farm		
Grasses	0.90 (0.24–3.11)	0.87
Forest	1.57 (0.41–5.63)	0.50
Bodies of water	2.97 (0.87–10.79)	**0.09**
Mountains	0.53 (0.15–1.92)	0.33
Regular flooding on property	3.06 (0.49–59.63)	0.31
Regular drought on property	0.42 (0.12–1.53)	0.18
Paved corral floor	0.50 (0.07–2.39)	0.42
Regular corral cleaning (weekly+)	0.66 (0.03–4.77)	0.72
Manure use for plant fertilization	1.25 (0.29–4.75)	0.75
Cultivated land	1.21 (0.36–4.26)	0.76
Pastures	0.61 (0.16–2.08)	0.44
Garden	2.40 (0.54–16.92)	0.30
Barn on property	0.59 (0.15–2.57)	0.45
Milk cows (dichotomous)	0.83 (0.11–4.04)	0.83
Meat cows (dichotomous)	0.40 (0.11–1.40)	0.15
Direct contact of horses with cattle	1.92 (0.35–9.34)	0.42
Contact of horses with cattle barn	2.30 (0.50–9.97)	0.27
Direct contact of chickens with cattle	1.53 (0.31–7.88)	0.60
Contact of chickens with cattle barn	1.39 (0.28–7.23)	0.69

Variable	OR (95% CI)	p-value
Animals slaughtered within 12 mo	1.12 (0.31–4.68)	0.87
Mechanism of milk ejection		
Calf contact	1.23 (0.16–6.59)	0.82
Conditioning	0.63 (0.11–4.96)	0.62
Other	Undefined	
Use of hand disinfection during milking	1.11 (0.30–4.16)	0.87
Use of milking line procedure	1.29 (0.17–26.74)	0.83
Use of udder disinfection during milking	1.04 (0.27–4.49)	0.96
Cattle fed after milking	0.20 (0.03–0.89)	**0.06**
Use of rodent control on the property	1.00 (0.27–3.47)	1.00
Previous knowledge of poxviruses	2.69 (0.61–18.89)	0.24
Animals on the property with history of vaccinia-like lesions	5.45 (1.28–37.89)	**0.04**
Presence of other animals on the farm:		
Horses	0.57 (0.03–3.98)	0.62
Chickens	2.04 (0.52–7.59)	0.29
Cats	0.56 (0.16–1.90)	0.36
Dogs	0.68 (0.14–2.66)	0.60
Pigs	1.21 (0.36–4.26)	0.76
Cows purchased, sold, or traded within the past 12 mo	1.06 (0.05–12.78)	0.96
Consumption of uncoiled milk	2.60 (0.30–17.81)	0.34
Type of cattle feed		
Pasture-fed	2.17 (0.26–14.61)	0.43
Commercial feed	0.28 (0.07–0.96)	**0.05**
Hay	1.00 (0.12–21.19)	1.00
Other food	4.06 (1.15–16.88)	**0.04**
Bulls shared for mating purposes	2.75 (0.72–13.61)	0.16
Frequent sightings in the past year of the following animals around cattle corral or pastures		
Rats	2.89 (0.82–11.97)	0.11
Mice	3.31 (0.92–13.87)	**0.08**
Opossums	Undefined	
Foxes	3.21 (0.50–63.12)	0.30
Bats	2.22 (0.49–9.20)	0.27
Monkeys	1.09 (0.26–5.65)	0.91
Snakes	2.36 (0.66–9.87)	0.20
Predatory birds	0.70 (0.20–2.55)	0.58
Humans with vaccinia-like lesions on the property	6.50 (1.11–124.09)	**0.09**

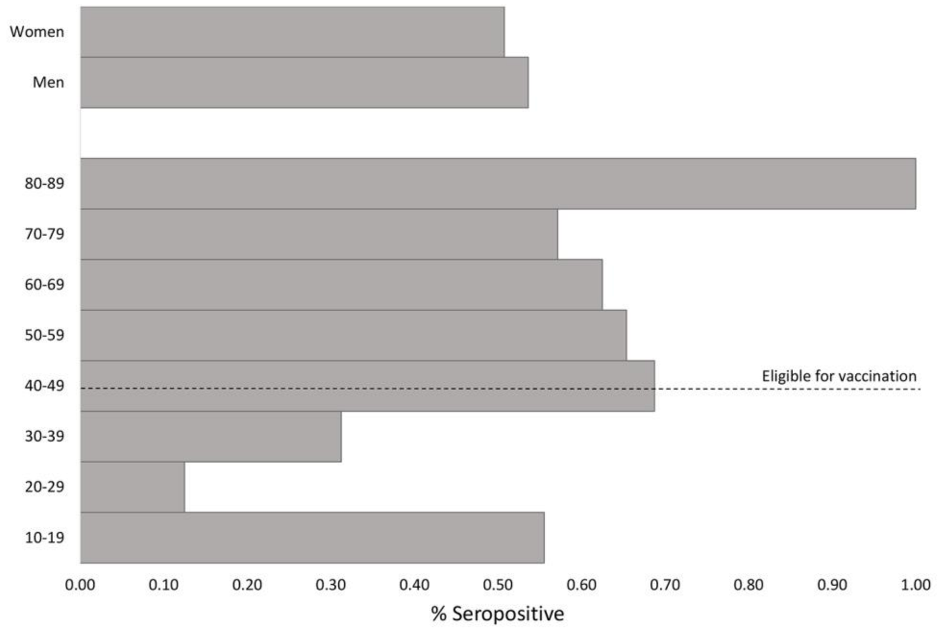

Appendix Figure 1. Anti-OPXV seropositivity, by age and sex. This graph demonstrates the distribution of seropositivity by decade and sex within the sampled population. The dotted line depicts the age of eligibility for prior smallpox vaccination.

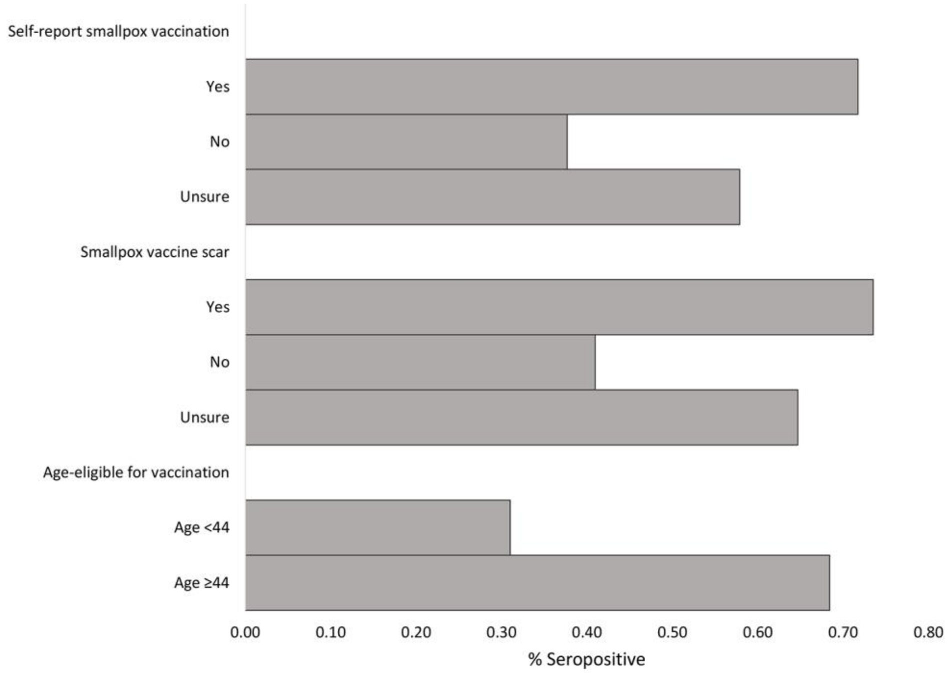

Appendix Figure 2. Anti-OPXV seropositivity, by smallpox vaccine indicators. This graph demonstrates the parameters that could serve as a proxy for prior smallpox vaccination and how this correlates with seropositivity.
